# Liberation of host heme by *Clostridioides difficile-*mediated damage enhances *Enterococcus faecalis* fitness during infection

**DOI:** 10.1128/mbio.01656-23

**Published:** 2023-12-11

**Authors:** Alexander B. Smith, Jonathan T. Specker, Katharine K. Hewlett, Troy R. Scoggins, Montana Knight, Abigail M. Lustig, Yanhong Li, Kirsten M. Evans, Yingchan Guo, Qianxuan She, Michael W. Christopher, Timothy J. Garrett, Ahmed M. Moustafa, Daria Van Tyne, Boone M. Prentice, Joseph P. Zackular

**Affiliations:** 1 Division of Protective Immunity, Children’s Hospital of Philadelphia, Philadelphia, Pennsylvania, USA; 2 Department of Chemistry, University of Florida, Gainesville, Florida, USA; 3 Department of Biomedical and Health Informatics, Children’s Hospital of Philadelphia, Philadelphia, Pennsylvania, USA; 4 Division of Infectious Diseases, Department of Medicine, University of Pittsburgh, Pittsburgh, Pennsylvania, USA; 5 Tsinghua University School of Medicine, Beijing, China; 6 Department of Pediatrics, Children’s Hospital of Philadelphia, Philadelphia, Pennsylvania, USA; 7 Department of Pathology, Immunology, and Laboratory Medicine, University of Florida, Gainesville, Florida, USA; 8 Department of Pathology and Laboratory Medicine, Perelman School of Medicine, University of Pennsylvania, Philadelphia, Pennsylvania, USA; 9 Institute for Immunology and Immune Health, Perelman School of Medicine, University of Pennsylvania, Philadelphia, Pennsylvania, USA; Universite de Geneve, Geneva, Switzerland

**Keywords:** *Clostridioides difficile*, *Enterococcus*, heme, infectious disease, gut microbiome, microbial ecology

## Abstract

Toxin production by *Clostridioides difficile* damages the colonic epithelium and leads to a robust inflammatory response. This disruption of the epithelial barrier markedly alters the nutritional landscape in the *C. difficile-*infected gut. The impact of toxin-mediated nutritional remodeling during *C. difficile* infection (CDI) on resident microbiota remains largely unexplored. One group of opportunistic pathogens, the enterococci, thrive during CDI, but it is unclear what strategies they employ to survive in this altered environment. Here, we demonstrate that *Enterococcus faecalis*, a heme auxotroph, takes advantage of *C. difficile* toxin-mediated damage to acquire host heme for enhanced fitness. Specifically, heme acquired from the *C. difficile-*infected gut is used by *E. faecalis* to populate a heme-dependent cytochrome and aerobically respire. This fitness advantage is specific to *C. difficile* toxin-mediated damage, as infection with a toxin-null strain of *C. difficile* does not provide *E. faecalis* with a fitness advantage. Finally, targeted disruption of the *E. faecalis* cytochrome (*cydABDC*) operon leads to a fitness defect in the *C. difficile-*infected gut. Together, this work demonstrates that *C. difficile* toxin remodels the gut ecosystem and improves the fitness of *E. faecalis* in a *cydABDC*-dependent manner. These data further highlight growing evidence of a cooperative partnership between *C. difficile* and enterococci that has implications on susceptibility to and severity of CDI.

**IMPORTANCE:**

*Clostridioides difficile* and *Enterococcus faecalis* are two pathogens of great public health importance. Both bacteria colonize the human gastrointestinal tract where they are known to interact in ways that worsen disease outcomes. We show that the damage associated with *C. difficile* infection (CDI) releases nutrients that benefit *E. faecalis*. One particular nutrient, heme, allows *E. faecalis* to use oxygen to generate energy and grow better in the gut. Understanding the mechanisms of these interspecies interactions could inform therapeutic strategies for CDI.

## INTRODUCTION

*Clostridioides difficile* is a Gram-positive toxigenic bacterium that causes a wide range of gastrointestinal pathologies and represents an urgent public health threat (1). The primary toxins of *C. difficile*, TcdA and TcdB, disrupt and damage the host epithelium and reshape the metabolic and nutritional landscape of the gut (2, 3). The mechanisms by which *C. difficile* toxins cause damage to the colonic epithelium are well established (4); however, it is unclear how toxin-mediated damage and subsequent reshaping of the nutritional landscape impact gut-resident microbiota. Recent studies have demonstrated that *C. difficile* infection (CDI) leads to increased levels of heme in the intestinal lumen in both mice and humans (5, 6). Heme is an essential cofactor for a variety of cellular processes (7, 8). Paradoxically, heme is also toxic to cells, and bacteria must sense and respond to toxic levels of heme (9). *C. difficile* encodes a heme efflux pump for efficient detoxification and co-opts heme to help combat oxidative stress during infection (5, 10). The impact of *C. difficile-*mediated heme influxes on the resident gut microbiota is not known.

Enterococci are members of the microbiota that have been shown to thrive in the *C. difficile*-infected gut (11–16). They represent both common human gut commensals as well as opportunistic pathogens that pose a serious risk to public health (17). Intrinsic and acquired antibiotic resistance coupled with a diverse metabolic repertoire allow enterococcal outgrowth in the gut during microbial community perturbation (12, 18, 19). Enterococcal domination often precedes translocation across the intestinal epithelium, leading to bacteremia and infection of distal body sites (18, 20). Our work has shown that outgrowth of enterococci enhances the virulence of *C. difficile* during infection (13). This work suggested that enterococcal outgrowth plays a central role in clinical outcomes of CDI; however, we still lack an understanding of the factors that promote enterococcal fitness in the *C. difficile*-infected gut. Opportunistic pathogens have been shown to leverage metabolic shifts in the gastrointestinal tract to gain a fitness advantage over commensal organisms (21–28). Specifically, inflammation-associated shifts in nutrient availability allow facultative anaerobes to aerobically respire and enhance growth (26–28). Nearly all facultative anaerobes encode a complete heme biosynthetic pathway in order to generate their own heme for respiration (29), enabling heme-containing cytochromes to establish a proton gradient and reduce molecular oxygen. Interestingly, enterococci are heme auxotrophs (7, 30) and must acquire heme exogenously to perform heme-dependent processes. *E. faecalis* encodes several heme-dependent enzymes, including a cytochrome *bd* terminal oxidase (encoded by the *cydABDC* operon), a catalase (KatA), a heme efflux pump (HatBA), and a heme-dependent transcriptional regulator (FhtR) (31–34). If supplied with exogenous heme, *E. faecalis* can perform aerobic respiration in the presence of oxygen by incorporating heme cofactors into the cytochrome subunits CydA and CydB. CydD and CydC are necessary for the assembly of the CydAB complex and were recently implicated as the elusive heme importers in *E. faecalis* (33, 35, 36). In the related pathogen *Streptococcus agalactiae*, also a heme auxotroph, respiration through a *cyd*-encoded cytochrome oxidase provides a fitness advantage in human blood (37). However, a functional *cydABDC* operon seems to impair the fitness of *E. faecalis* in the bloodstream by sensitizing it to the oxidative burst of immune cells (34, 38). These findings suggest that the conserved *cydABDC* operon may be beneficial to *E. faecalis* in its native gastrointestinal tract environment but may be detrimental when the bacteria infect extraintestinal body sites.

CDI results in dysanaerobiosis at the epithelial barrier (39), but it remains unclear whether altered oxygen availability and toxin-dependent liberation of heme support a permissive environment for enterococci to perform aerobic respiration and thrive in the gut. In this study, we demonstrate that heme influx during CDI provides a fitness advantage to *E. faecalis*. This effect is dependent on *C. difficile* toxin and specific to CDI-associated inflammation. We propose that *C. difficile*-mediated damage supports polymicrobial cooperation with the enterococci that exacerbates disease caused by co-infection with these two pathogens.

## RESULTS

**Fig 1:**
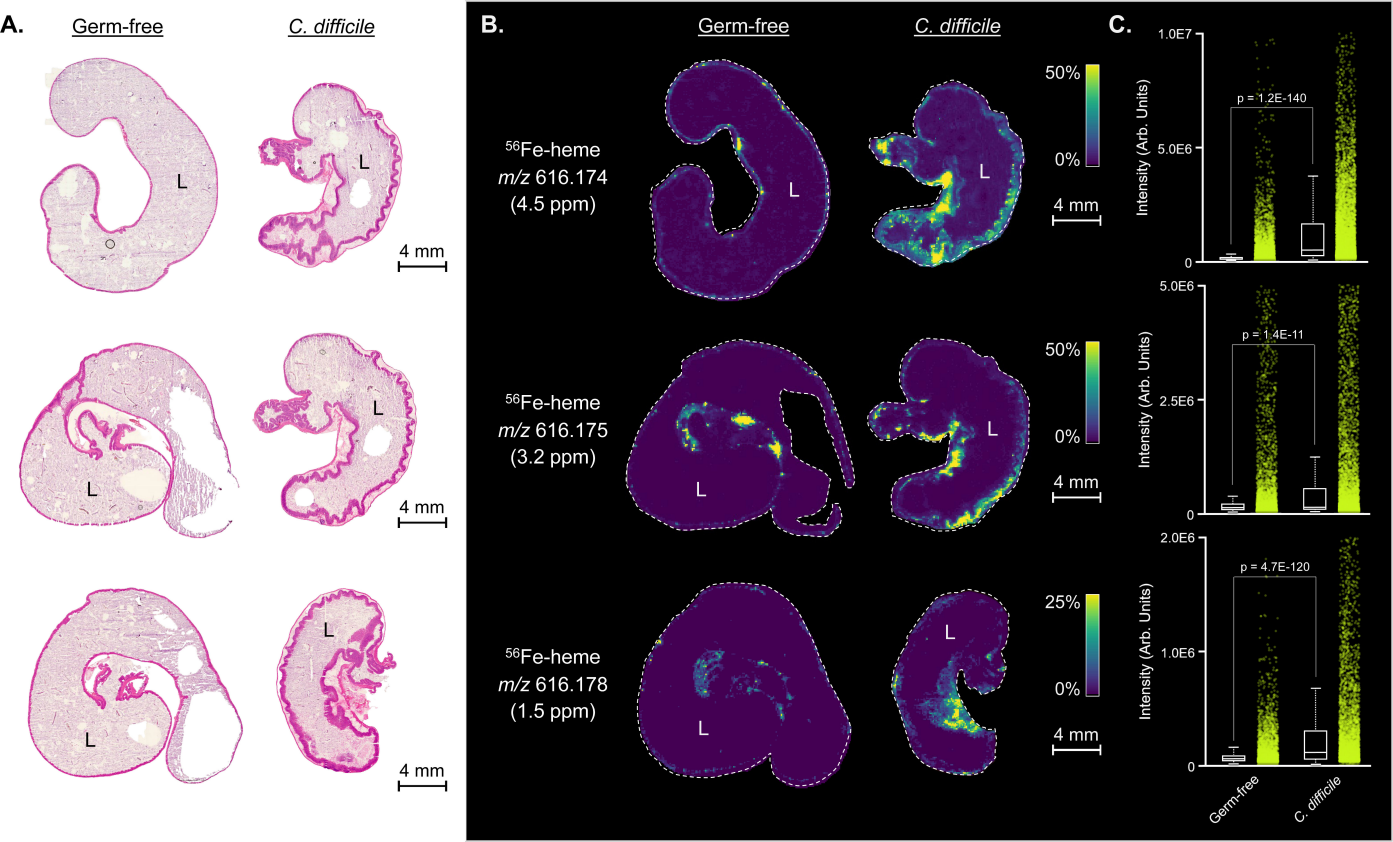
(**A**) Hematoxylin and eosin-stained cecal tissue sections from germ-free and *C. difficile* (CD196) mono-infected mice (*n* = 3 tissue replicates). L marks the lumen of the cecum. (**B**) Matched MALDI imaging mass spectrometry ion images of ^56^Fe-heme displaying heme localization to the mucosa of the infected intestinal tract. (**C**) Relative quantitation of MALDI imaging mass spectrometry ^56^Fe-heme intensities between germ-free and *C. difficile* (CD196) mono-infected mice.

Damage and inflammation associated with CDI leads to a remodeling of the nutritional landscape in the gut. Specifically, heme is highly abundant in the stools of patients with CDI, and hemoglobin is enriched in the cecum of mice infected with *C. difficile* (5, 6). We sought to confirm that the influx of heme into the tissues and lumen of the gut was directly associated with *C. difficile*-mediated damage. Using germ-free mice infected with *C. difficile*, we spatially mapped heme with matrix-assisted laser desorption/ionization (MALDI) imaging mass spectrometry. As opposed to uninfected germ-free mice, mice mono-infected with *C. difficile* showed an influx of heme into the lumen and surrounding tissue during CDI (Fig. 1). These data demonstrate that *C. difficile*-mediated damage, independent of endogenous microbiota, increases available heme in the lumen of the gut.

**Fig 2:**
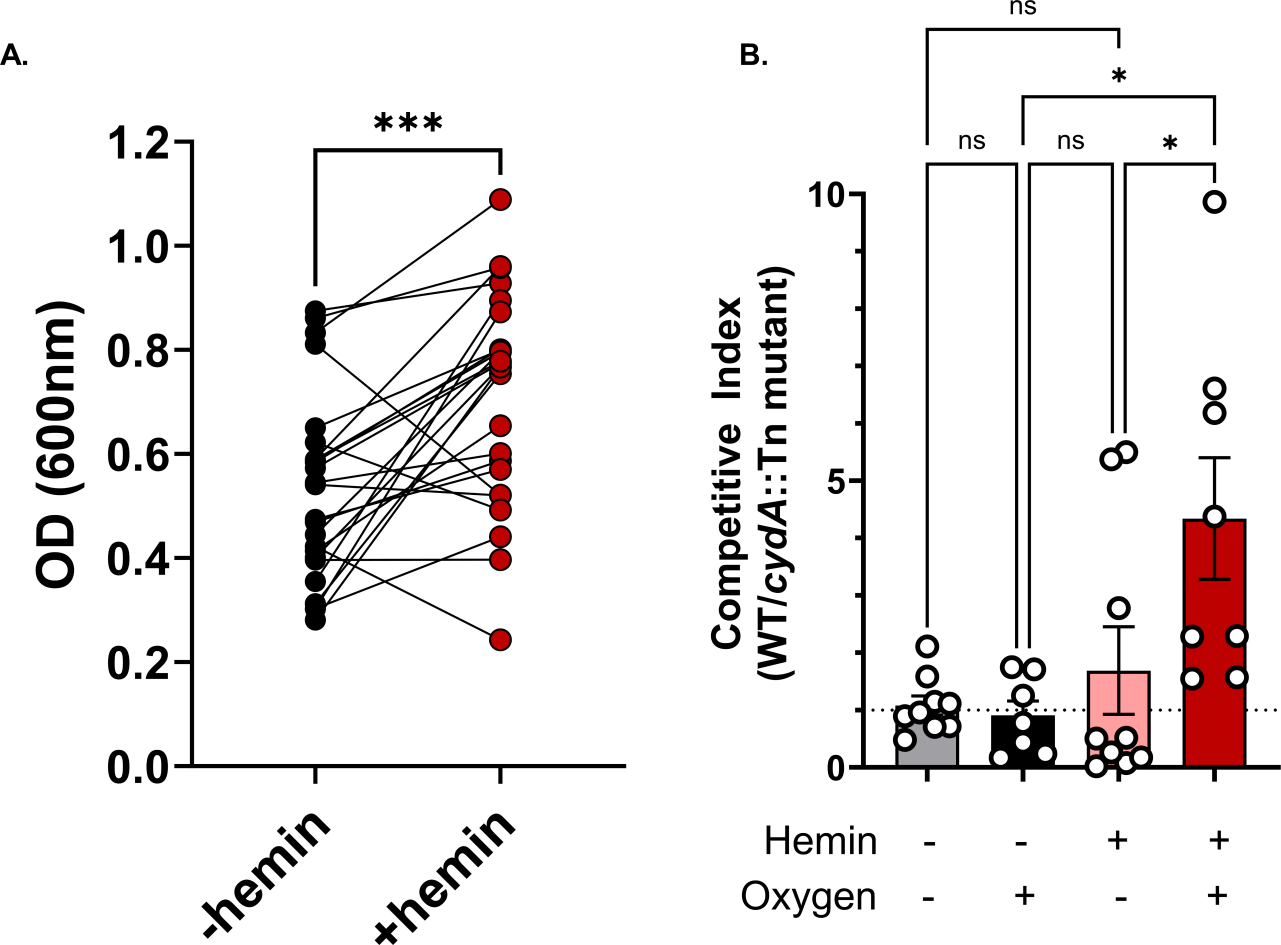
(**A**) *E. faecalis* isolate growth after overnight aerobic culture at 37°C in the absence (–hemin) or presence (+hemin) of 10 uM hemin. OD600 values are averaged from three replicate measurements and lines connect measurements from the same isolate. *N* = 25; significance was measured by a paired *t*-test, *** (*P* < 0.001). (**B**) Competitive index of wild-type *E. faecalis* OG1RF relative to *E. faecalis cydA*::Tn *in vitro* after 24 hours of growth in the indicated conditions as measured by selective CFU plating. Data are mean +/− SEM; *n* = 7. No hemin/aerobic, *n* = 8 hemin/aerobic, *n* = 9 for all other groups; Kruskal-Wallis test for significance. ns = not significant, * (*P* < 0.05).

The role that heme plays in shaping the ecosystem and ecology of the gut microbiota during CDI has not been explored. Enterococci are of particular interest, as they play a critical role in shaping *C. difficile* behavior and virulence (13). We first sought to establish whether the capacity for heme utilization was prevalent in clinical strains of enterococci isolated from adult patients with CDI. We isolated 25 *E. faecalis* strains from stool samples collected from patients with CDI and grew them in aerobic culture with or without supplemental hemin. About 80% of these CDI-associated *E. faecalis* strains showed enhanced growth in the presence of hemin and oxygen (Fig. 2A). Next, we sought to determine if increased *E. faecalis* fitness in the presence of heme was dependent on the *cydAB* cytochrome. Using a transposon mutant with a disrupted *cydA* (OG1RF_11666) gene (*cydA*::Tn), which encodes the subunit containing two heme prosthetic groups, we performed *in vitro* competition assays with the wild-type OG1RF *E. faecalis* strain. These experiments demonstrated that *cydA* confers a fitness advantage for *E. faecalis,* but only in the presence of both hemin and oxygen (Fig. 2B). This confirms past reports that *E. faecalis* has the capacity to perform aerobic respiration when supplied with heme and oxygen—conditions experienced in the CDI gut (39).

**Fig 3:**
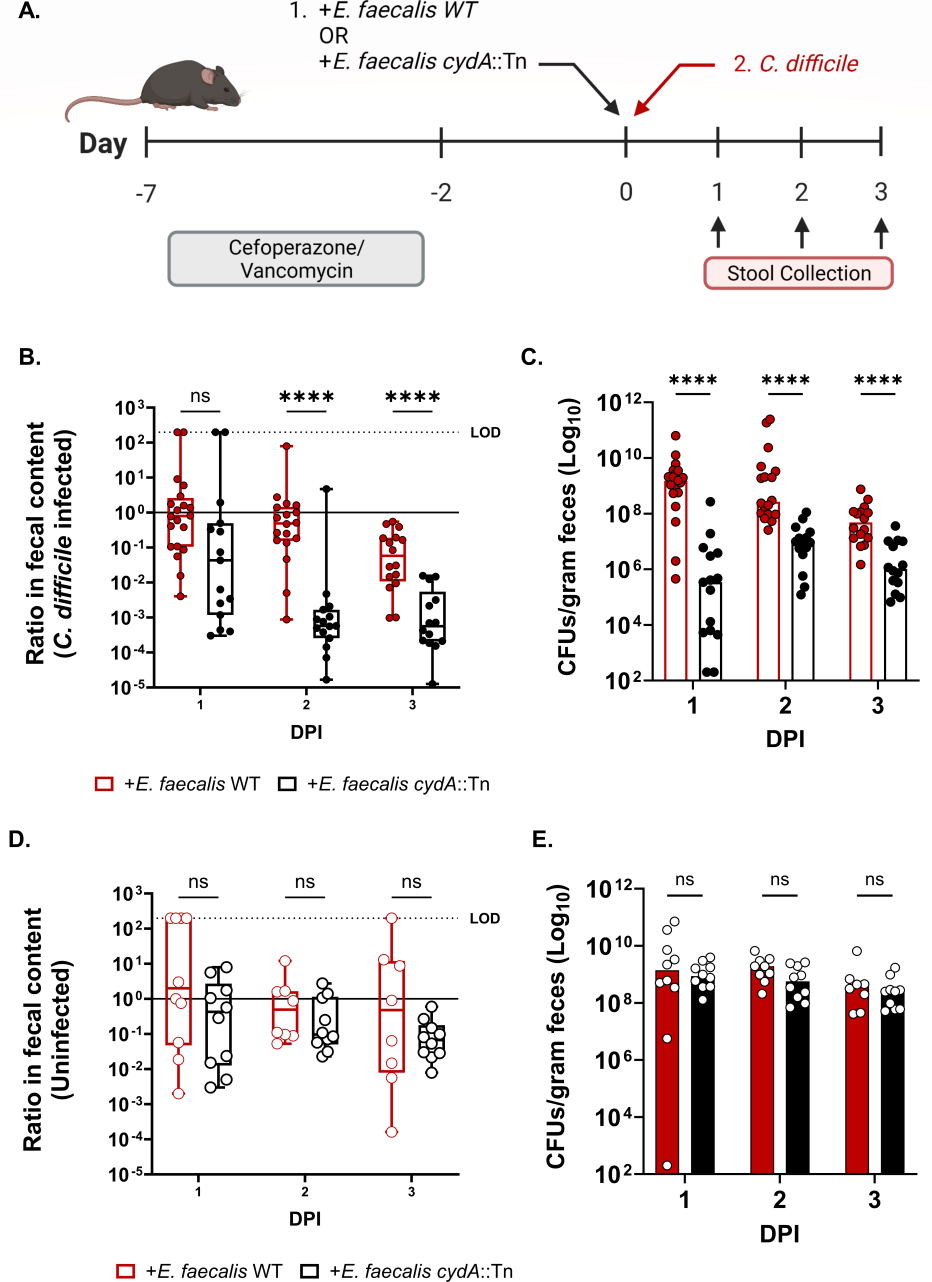
(A) Schematic of *C. difficile* mouse infection model. (B) Ratio of *E. faecalis* OG1RF or *E. faecalis cydA*::Tn CFU burdens to endogenous enterococci CFU burdens in the stool of mice treated with cefoperazone and vancomycin and gavaged with each exogenous *E. faecalis* strain immediately prior to *C. difficile* infection (CDI), and (C) CFUs of each exogenously introduced *E. faecalis* strain in *C. difficile*-infected mice. Data are shown with median; *n* = 20 mice per day for CDI/*E. faecalis* WT; *n* = 15 mice per day for CDI/*E. faecalis cydA*::Tn. *P-*values are from multiple Mann-Whitney tests for significance with Bonferroni-Dunn correction for multiple comparisons. (D) Ratio of *E. faecalis* OG1RF or *E. faecalis cydA::*Tn CFU burdens to endogenous enterococci CFU burdens in mice similarly treated as above but without CDI, and (E) CFUs of each exogenously introduced *E. faecalis* strain in the uninfected mice. Data are shown with median; *n* = 10 per group per day for no CDI; *P*-values are from multiple Mann-Whitney test for significance with Bonferroni-Dunn correction for multiple comparisons. ns = not significant, **** (*P* < 0.0001). LOD = limit of detection. Box plots show minimum, maximum, median, and interquartile range. DPI = days post-infection.

Next, to explore whether the *cydABDC* operon confers a fitness advantage to *E. faecalis* during CDI, we co-infected conventional mice with *C. difficile* and either wild-type *E. faecalis* or *E. faecalis cydA*::Tn. Mice were first transiently depleted of their endogenous enterococcal population using vancomycin prior to colonization with *E. faecalis*. Mice were then infected with *C. difficile* and the endogenous and exogenous populations of enterococci were tracked over the course of infection (Fig. 3A). Ratios of exogenous *E. faecalis* to endogenous enterococci were calculated to quantify the fitness of each exogenous strain. Wild-type *E. faecalis* significantly outperformed *E. faecalis cydA*::Tn during the course of CDI, with the *cydA* mutant being rapidly cleared from the CDI gut (Fig. 3B). The mutant strain was also present at a significantly lower absolute abundance during the time course of infection compared to the wild-type strain (Fig. 3C). Notably, CydA was not required for *E. faecalis* fitness in the absence of CDI, suggesting a specific role for this cytochrome during CDI (Fig. 3D and E). In either case, there was no significant difference in the endogenous enterococcal population between mice colonized with wild-type *E. faecalis* or *E. faecalis cydA*::Tn (Fig. S1C and D). In addition to the cytochrome, *E. faecalis* encodes a heme-dependent catalase (*katA,* OG1RF_11314). Surprisingly, a transposon mutant lacking catalase function (*katA*::Tn) does not exhibit a defect during CDI or in healthy mice (Fig. S1B and S2). Together, these data suggest that *E. faecalis* uses host heme to perform aerobic respiration in CDI gut and gains a competitive advantage against other members of the microbiota.

**Fig 4:**
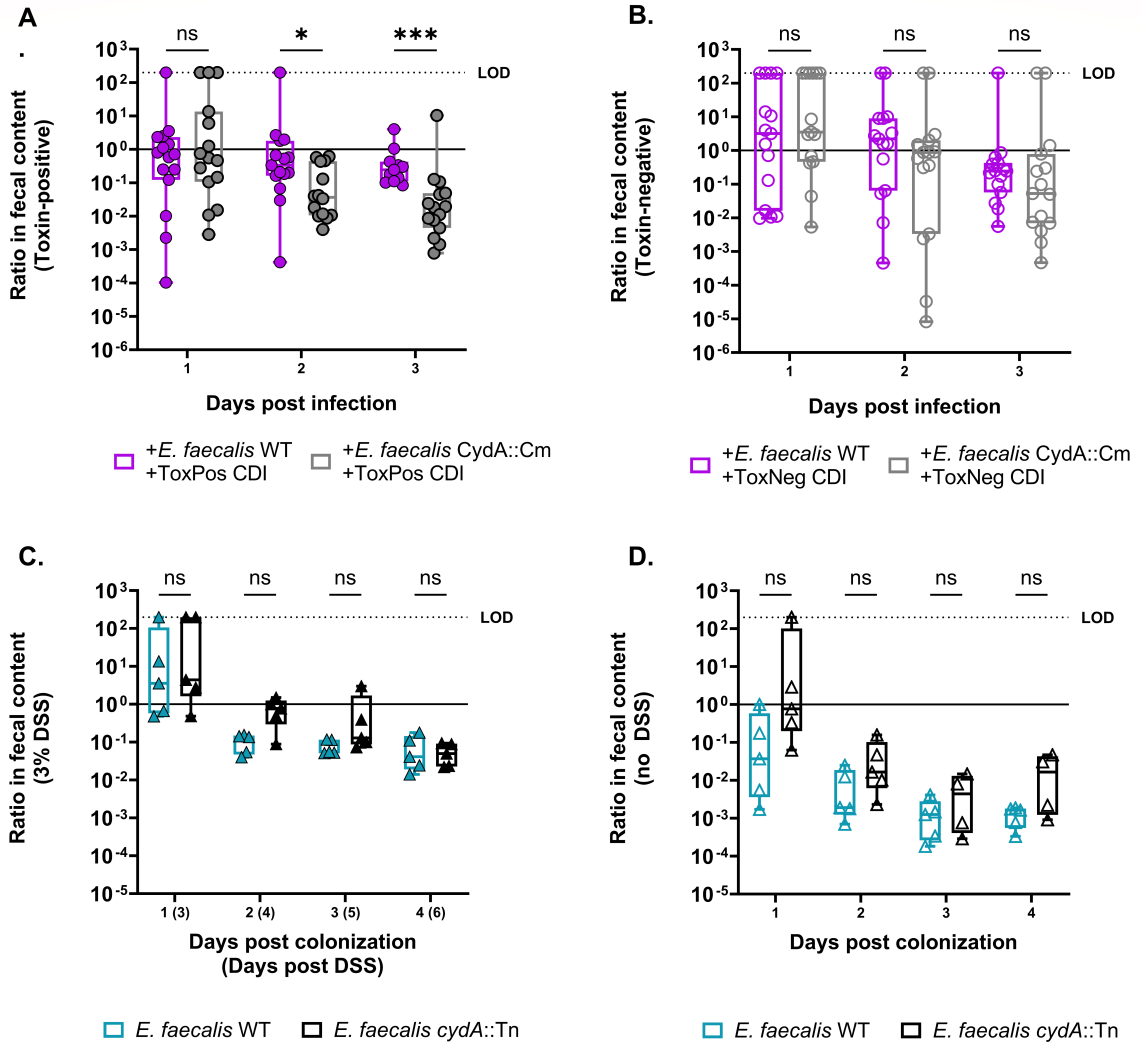
(A) Ratio of *E. faecalis* OG1RF or *E. faecalis cydA*::Tn CFU burdens to endogenous enterococci CFU burdens in the stool of mice treated with cefoperazone and vancomycin and gavaged with each exogenous *E. faecalis* strain immediately prior to infection with *C. difficile* M7404 *tcdA^+^tcdB ^+^* (toxin-positive) or (B) *C. difficile* M7404 *tcdA^−^tcdB*^−^ (toxin-negative). Data are shown with median; *n* = 15 mice per group per day; multiple Mann-Whitney test for significance with Bonferroni-Dunn method for correction for multiple comparisons, * (*P* < 0.05), *** (*P* < 0.001). (C) Ratio of *E. faecalis* OG1RF or *E. faecalis cydA*::Tn CFU burdens to endogenous enterococci CFU burdens in the stool of mice treated with vancomycin and gavaged with each exogenous *E. faecalis* strain with 3% DSS treatment (D) or without. Data are shown with median; *n* = 5 mice per group per day; multiple Mann-Whitney test for significance with Bonferroni-Dunn method for correction for multiple comparisons. ns = not significant. LOD = limit of detection. Box plots show minimum, maximum, median, and interquartile range. DPI = days post-infection.

Next, to determine if this phenomenon was dependent on the action of the *C. difficile* toxins, we performed mouse infections with *C. difficile* strains incapable of producing toxin. Mice were infected with either a toxin-mutant strain of *C. difficile* M7404 (*tcdA^−^tcdB*^−^) or a toxin-positive strain (*tcdA*^+^*tcdB*^+^). M7404 and CD196 are ribotype 027 strains of *C. difficile* and induce a similar level of disease pathology during infection (Fig. S3). We found that the *E. faecalis cydA*::Tn mutant only suffered a fitness defect when the *C. difficile* toxin was present (Fig. 4A and B), suggesting that toxins are critical to liberating heme and enabling *cydA*-specific advantages to *E. faecalis* in the CDI gut. We hypothesized that other inflammatory conditions would release heme and cause a similar defect in the *cyd* mutant. We measured heme by ultra-high-performance liquid chromatography coupled with high-resolution mass spectrometry (UHPLC-HRMS) in the stool of mice infected with toxin-positive *C. difficile* or toxin-negative *C. difficile*, or mice treated with 3% dextran sodium sulfate (DSS). We found elevated levels of heme in both the toxin-positive CDI and DSS conditions (Fig. S4A). Notably, the advantage conferred by the *E. faecalis* cytochrome appears to be relatively specific to CDI-associated damage and inflammation as chemically induced colitis through DSS treatment did not lead to a fitness disadvantage for the *cydA*::Tn mutant (Fig. 4C and D). While aerobic respiration appears to play an important role for *E. faecalis* during CDI, these data suggest other factors may shape *E. faecalis* fitness in the context of chemically induced colitis. Interestingly, metabolomic data from human patients shows that fecal heme levels are significantly higher in children with IBD + CDI compared to IBD alone (Fig. S4B), suggesting that the role of heme during CDI is clinically relevant. Taken together, these results demonstrate that *C. difficile* toxin-mediated damage provides a fitness advantage for *E. faecalis* through heme liberation and that this phenomenon is not generalizable to all inflammatory conditions.

Finally, to specifically determine the conservation of heme utilization in gut isolates of enterococci from CDI patients, we analyzed the genomes of strains of *E. faecalis* described above. Whole-genome sequencing revealed a diverse repertoire of virulence factors, antibiotic resistance markers, and plasmids that varied widely between strains. This indicates that there is a diversity of genetic and phenotypic properties in the enterococcal populations across CDI patients. However, all strains that were sequenced encoded a fully intact *cydABDC* operon (Fig. S5), suggesting that the capacity to use heme as a resource in the CDI gut is widely conserved. These data demonstrate that the ability to use heme to gain a fitness advantage in the CDI gut is widely conserved across *E. faecalis* strains found in this perturbed ecosystem.

## DISCUSSION

Of the many changes in the gut ecosystem observed during acute CDI, one of the most significant is the increase in opportunistic pathogens in the intestinal microbiome (40). One of the most clinically relevant and important members of the CDI-associated microbiota are the enterococci (13). The mechanisms by which enterococci, particularly *E. faecalis*, thrive in the CDI gut environment remain unexplored. In this study, we demonstrate that toxin production by *C. difficile* reshapes the gastrointestinal ecosystem and provides a relative fitness advantage to *E. faecalis*. Specifically, toxin-mediated damage liberates heme, which *E. faecalis* incorporates into its cytochrome to perform aerobic respiration. These observations provide an important mechanistic paradigm for the cooperative interactions between these two important and commonly co-occurring pathogens.

Bacterial pathogens leverage changes in the nutritional landscape of the large intestine during inflammation to gain a fitness advantage. For example, the Enterobacteriaceae, a family of commensals and opportunistic pathogens, outcompete commensal bacteria when inflammation and damage liberate alternative electron acceptors into the gut environment (22–24, 27). Specifically, *Escherichia coli* takes advantage of the altered nutrient pool to respire oxygen, which is known to be present in the gut during DSS treatment (26, 41). Moreover, both *Citrobacter* and *Salmonella* produce toxins that disrupt the intestinal epithelium and allow elevated levels of oxygen into the lumen that they can then use to respire (22–24, 27). CDI and toxin production are associated with the release of nutrients such as heme and amino acids into the lumen, as well as disruption of the hypoxic barrier that normally maintains strict anaerobicity in the large intestine (2, 5, 6, 13, 39).

The large intestine is anaerobic in healthy individuals, preventing aerobic respiration and thereby supporting anaerobes that promote homeostasis and host health. Under inflammatory conditions, epithelial damage leads to a PPARγ-dependent shift in colonocyte metabolism that allows molecular oxygen to diffuse into the lumen of the gut (41). Combined with oxygen delivered directly from an influx of red blood cells, the large intestine enters a state of dysanaerobiosis alongside the dysbiosis of the microbiota (24, 42). It has also been shown directly that damage and dysbiosis caused by the *C. difficile* toxins eliminate the hypoxic barrier in the epithelium and allow oxygen into the gut lumen where it can be utilized by respiring bacteria (39). Together this likely creates an environment conducive to expansion by *E. faecalis*, but only in the presence of heme (13).

**Fig 5:**
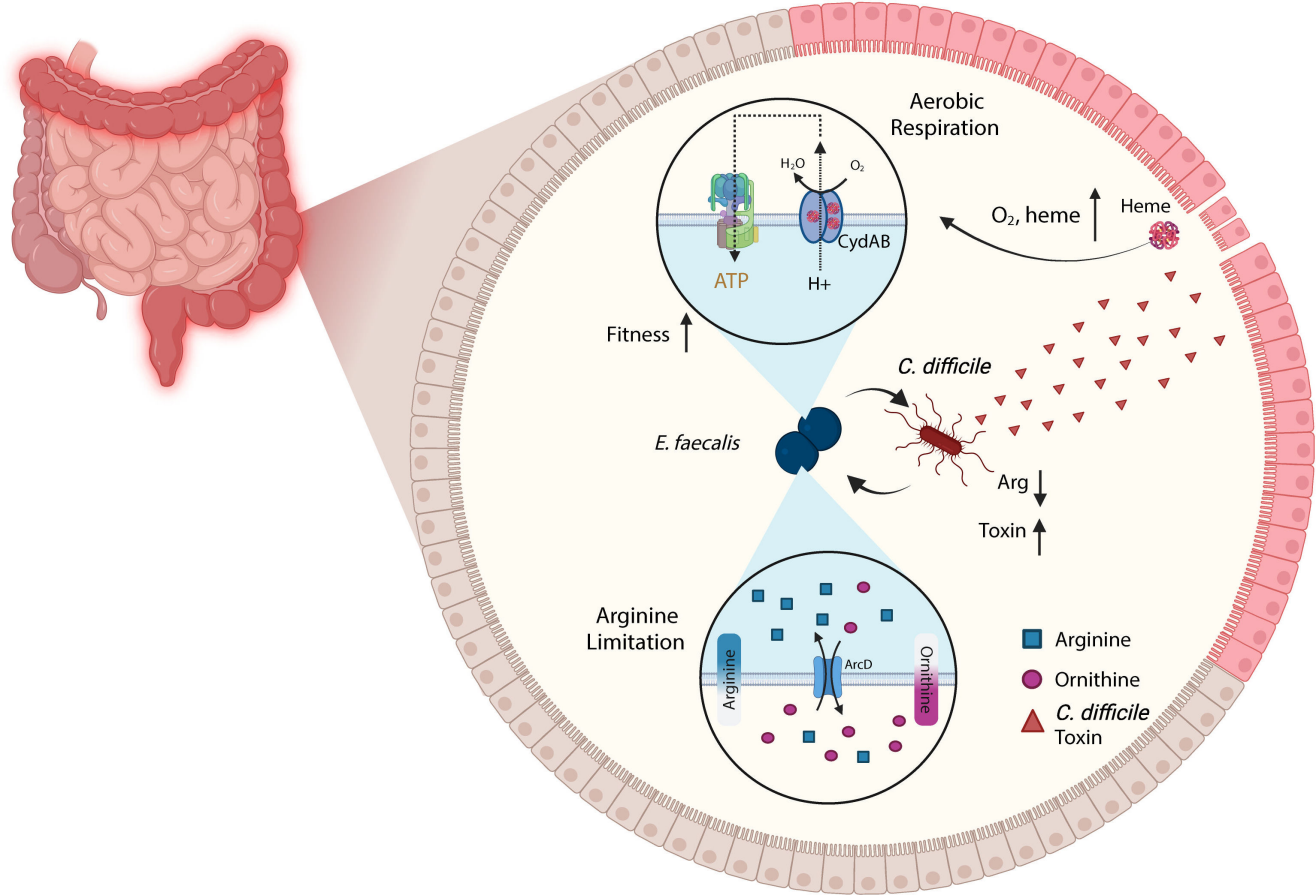
Model schematizing known interactions between *Enterococcus faecalis* and *Clostridioides difficile*. As described in this study, *C. difficile* toxins cause damage to the host that releases heme and oxygen into the lumen. *E. faecalis* uses heme and oxygen to respire and thrive through the *cydABDC* operon (top). Previous work describes a relationship wherein *E. faecalis* consumes and limits the luminal arginine pool. This arginine restriction acts as a reversible cue that causes *C. difficile* to increase toxin production, forming a positive ecological feedback loop that exacerbates disease severity (bottom).

*E. faecalis* is relatively unique in that its capacity for aerobic respiration is limited by heme auxotrophy. As suggested in other studies and explicitly here, the *C. difficile* toxins are sufficient to release host heme into the gut lumen (5, 6). Enabling a fitness advantage to endogenous *E. faecalis* could lead to a host of complications during CDI. For example, we previously showed that enterococci exacerbate CDI by dramatically remodeling the metabolic environment in the gut and consuming luminal arginine (Fig. 5) (13). This acts as a reversible cue to *C. difficile* to increase toxin production and ultimately leads to increased pathogenesis. Additionally, *E. faecalis* and *C. difficile* form robust interspecies biofilms that are protected from antibiotic treatment and could act as a reservoir for persistence and recurrence (13). Moreover, heme utilization in these tight associations enables metronidazole resistance in *C. difficile* (43, 44). In each case, the ability of *C. difficile* to support the fitness of *E. faecalis* could further enhance these pathogenic phenotypes. *Staphylococcus aureus* and *E. coli* have been shown to directly cross-feed heme to *E. faecalis* and bolster its growth *in vitro* (31, 36). In fact, prior reports showing that respiration actually handicaps *E. faecalis* within the hemoglobin-rich environment of the bloodstream, along with interspecies biofilms allowing for the robust exchange of heme, support the idea that this functionality evolved for use in the polymicrobial environment of the gut (36, 38).

The precise origin of heme utilized by *E. faecalis* in the context of CDI remains to be discovered. *E. faecalis* can directly import heme or first degrade hemoglobin and other hemoproteins using its encoded gelatinase (31, 36). However, the ability of *E. faecalis* to derive heme cofactors from erythrocytes varies across isolates. Hemolysis relies on the cytolysin encoded by the *cyl* operon (45, 46). Since the strain used in this study, OG1RF, does not encode the cytolysin, it cannot lyse erythrocytes and access heme without assistance from *C. difficile*. However, clinical isolates of *E. faecalis* variably encode the cytolysin (Fig. S5). Therefore, the genotype of *E. faecalis* might impact its ability to use heme and could determine the ecological scenarios under which *E. faecalis* gains a fitness advantage *in vivo*.

Though the nutritional requirements to perform aerobic respiration are similarly met in the DSS-treated mice, *E. faecalis* does not suffer a disadvantage from losing *cydA*. This would imply that *E. faecalis* may prioritize other modes of metabolism in this environment. The nutritional state of the gut changes dramatically after DSS treatment (47); however, these data demonstrate that different drivers of inflammation can have distinct impacts on the ecology of the microbiome. The complex relationship between the gut environment and *E. faecalis* fitness warrants further study.

In our study we did not observe a defect in a KatA-deficient strain of *E. faecalis* during CDI. *E. faecalis* encodes three heme-independent peroxidases that could compensate for the activity of KatA (39, 48, 49). It is possible that the two major heme-dependent complexes of *E. faecalis*, CydABDC and KatA, also synergize during CDI to provide a fitness advantage. During infection, reactive oxygen species (ROS) are produced at the epithelial interface by the host NADPH oxidase NOX1 as a defense mechanism against invading pathogens. *E. coli* was shown to convert these ROS into molecular oxygen via its catalases and then undergo respiration through a cytochrome *bd* oxidase (28). With heme supplied by the host during CDI, we suspect that *E. faecalis* could similarly convert ROS from NOX1/2 via KatA into oxygen for respiration via CydABDC. Moreover, the action of the catalase may not directly benefit *E. faecalis*, but could provide cross-protection to other microbes that benefit *E. faecalis* ecologically (50). Further studies are required to fully understand the tripartite interactions between these two pathogens and the host as well as the role of heme in the oxidative stress response of *E. faecalis*.

The work described in this study details an indirect route by which *C. difficile* is able to cross-feed heme to *E. faecalis*. This is also the first reported instance of the *cydABDC* operon boosting *E. faecalis* fitness within its host. Even though aerobic respiration seems to impair *E. faecalis* in the bloodstream (34), the ability to grow to high levels in the gut could potentially allow translocation of heterogenous strains, thereby increasing the risk of enterococcal complications such as bacteremia during CDI. Further studies are required to understand the consequences of this fitness advantage during CDI as well as infections or conditions beyond CDI that enable *E. faecalis* in this manner. Additionally, this study establishes that the action of the *C. difficile* toxins remodels the gut environment and provides altered nutrient niches for members of the gut microbiota, opening a broad avenue of ecological interactions during infection. Understanding the ecology between these two pathogens is critical to the development of effective treatment strategies for patients suffering from CDI.

## MATERIALS AND METHODS

### Bacterial strains and growth conditions

*C. difficile* and *E. faecalis* strains were grown at 37°C in an anaerobic chamber (90% nitrogen, 5% hydrogen, 5% carbon dioxide; Coy Lab Products) or aerobically in brain-heart-infusion broth (BD Life Sciences) supplemented with 0.5% yeast extract (BD Life Sciences) and 0.1% L-cysteine (Sigma-Aldrich) (BHIS) unless otherwise stated. Media was supplemented with 50 µM hemin (Sigma-Aldrich) when noted. Strains are listed in Table S1. All strains were confirmed by whole genome sequencing. The location of the transposon was validated in each transposon mutant used in the study, and we confirm no significant off-target mutations (Fig. S1A).

### *In vitro* competition assay

Wild-type *E. faecalis* and *E. faecalis cydA*::Tn overnight cultures were mixed at a 1:1 ratio and subcultured at a 1:100 ratio into fresh BHIS media. Mixed cultures were grown under the indicated conditions for 24 hours. Abundance of each strain was quantified as colony-forming units (CFUs) on BHIS agar and BHIS + chloramphenicol (10 µg/mL) agar. The ratio of output was normalized to the ratio of inoculum to quantify competitive index.

### Animal models of infection

Animal experiments were approved by the Animal Care and Use Committees of the Children’s Hospital of Philadelphia (IAC 18–001316). For CDIs in conventional facilities, 4- to 8-week-old C57BL/six male mice were purchased from Jackson Laboratories and given one week to equilibrate their microbiota prior to experimentation. All experimental manipulations were performed in a biosafety level two laminar flow hood. Mice were housed in individual cages under the same conditions during the experiment, and all mice were culture-negative for *C. difficile* prior to infection. For all CDIs, mice were given antibiotics (0.5 mg/mL cefoperazone + 1 mg/mL vancomycin) in drinking water *ad libitum* for 5 days followed by a 2-day recovery period and subsequent infection. Mice were confirmed culture-negative for endogenous enterococci after vancomycin treatment via selective plating as described below. Mice were infected with 5 × 10^8^ *E. faecalis* (wild-type strain OG1RF, *cydA*::Tn, or *katA*::Tn) cells. *E. faecalis* cells were grown to stationary phase, washed in cold PBS prior to infection, and orally gavaged. Mice were subsequently co-infected via oral gavage with 1 × 10^5^ spores of *C. difficile* resuspended in sterile PBS. *C. difficile* strains CD196, M7404, and M7404 TcdA^−^ TcdB^−^ were used for conventional infections, as described in the text. Mice were monitored for survival and were euthanized after reaching a terminal endpoint of appearing moribund or experiencing weight loss >20% from baseline. *C. difficile* and enterococcal CFUs were quantified daily from fecal samples. All samples were collected, and all stool-related data are reported, unless animals were too sick to acquire a fresh stool sample. Samples were diluted and homogenized in PBS and serially plated onto taurocholate cycloserine cefoxitin fructose agar (TCCFA) for *C. difficile* and Bile Esculin agar for total enterococci. *E. faecalis* OG1RF strains were grown on Bile Esculin agar with rifampicin (200 µg/mL).

For infections involving dextran sodium sulfate (DSS), mice were given vancomycin (1 mg/mL) in drinking water *ad libitum* for 5 days followed immediately by 3% DSS in drinking water *ad libitum* for 2 days. Mice were infected by oral gavage with 5 × 10^8^ *E. faecalis* (wild-type strain OG1RF or *cydA*::Tn) cells as described above. Mice were continued on the course of DSS throughout the infection and were similarly monitored for survival. Enterococcal CFUs were quantified daily from fecal samples as described above.

### Imaging mass spectrometry

Tissue samples to be analyzed by MALDI imaging mass spectrometry were embedded in a 20% mixture (vol/vol) of optimal cutting temperature (OCT) compound and water, shipped to the University of Florida on dry ice, and stored at −80°C until analysis. Tissue sections were prepared at 12 µm thickness using a Leica CM 3050S Research Cryostat (Leica Biosystems) (−30°C object temperature, −28°C chamber temperature) and thaw-mounted onto indium tin oxide-coated microscope slides. Samples to be compared via imaging mass spectrometry were mounted on the same microscope slide to ensure identical sample preparation and facilitate accurate analyte comparisons between tissue types. Slides with mounted tissue sections were then warmed to room temperature in a desiccator for ~30 minutes before application of a 2,5-dihydroxybenzoic acid (DHB; Sigma Aldrich) MALDI matrix solution (50% methanol, 0.1% TFA) using a TM-sprayer (HTX Technologies) (51). The robotic spraying conditions were velocity 1,200 mm/minutes, flow rate 0.1 mL/minutes, spray temperature 85°C, heated tray temperature, 38°C, number of passes 6, track spacing 3 mm, and sheath gas pressure 10 psi.

All imaging mass spectrometry experiments were performed in positive ion mode on a 7T Fourier transform ion cyclotron resonance (FT-ICR) solariX mass spectrometer equipped with a dynamically harmonized ParaCell (Bruker Daltonics). The instrument contained an Apollo II dual MALDI/ESI source that uses a Smartbeam II Nd:YAG MALDI laser (2 kHz, 355 nm). Images were acquired at a pixel spacing of 125 µm in both the x and y dimensions using a ∼25-µm laser beam and a 110-µm Smart Walk (300 laser shots). Tissue data were collected from m/z 200 to 1,200 using a 0.9787 s time-domain transient length, resulting in a resolving power of ∼87,000 (FWHM at m/z 598). Internal calibration using a quadratic fit was performed using common endogenous lipid ions. Ion image distributions and intensity box plots were visualized using SciLS Lab software (Bruker Daltonics). Ion images are displayed without normalization and using pixel interpolation. Ion intensity box plots were generated by extracting .imzML files and converted to .csv format using python version 3.10.9. The statistical analysis was performed using the SciPy module in Python version 3.10.9 (52). *P*-value was calculated by comparing the t-statistic of the ion intensity data against a theoretical t-distribution. Following image acquisition, serial tissue sections were stained using hematoxylin and eosin (H&E), bright field scanned using a Axio Imager M2 Microscope (Carl Zeiss Microscopy), and visualized using Zen microscopy software (Carl Zeiss Microscopy).

### Ultra-high-performance liquid chromatography coupled with high-resolution mass spectrometry

Mouse stool was homogenized in cold PBS, and the supernatant was filter sterilized using a 0.2-µm filter. The sterile supernatant was extracted utilizing a neutral acetone extraction (53). All solvents used for sample preparation were Fisher HPLC grade. Briefly, 800 µL of ice-cold acetone was added to 100 µL of sterile supernatant. The sample and acetone mixtures were vortexed and allowed to rest on ice for 15 minutes, vortexed again, and allowed to rest for another 15 minutes for a total of 30 minutes of rest. The mixture was then centrifuged for 10 minutes at 4°C and 3,260 × *g* to pellet the proteins. Eight hundred microliter of the supernatant was taken and dried in a microcentrifuge tube under a nitrogen gas stream at room temperature. Dried supernatants were then reconstituted using 100 µL of 25% methanol/75% 0.1% formic acid water and allowed to solubilize for 30 minutes, with vortexing every 15 minutes.

Samples and blanks were analyzed on a UHPLC-HRMS system consisting of a Dionex UltiMate 3000 UHPLC (Thermo Fisher Scientific) coupled to a Q Exactive Orbitrap Mass Spectrometer (Thermo Fisher Scientific). An Avantor ACE C18-PFP column (Catalogue number: EXL-1010–1002U) placed in a column oven set to 50°C was used for separation with a gradient elution method using water with 0.1% formic acid (FA) and methanol as mobile phases A and B, respectively. All solvents used for LC separations were Fisher Optima grade. All analyses were acquired in positive electrospray mode (Table S2).

Inclusion of integrated peak values into the post-processing data analysis was contingent on the following criteria being met: matching peak retention time (within 0.05 minutes of chemical standard), matching the exact mass of the most abundant isotope (5 ppm error 616.1767 ± 0.0031 with respect to theoretical), at least eight scans across the peak at full width at half max (FWHM), and visually matching isotopic distribution with respect to theoretical spectrum. Data analysis was performed using Qual Browser from Thermo Xcalibur (4.3.73.11). Integration was performed using boxcar smoothing with five points, a mass tolerance of 10 ppm, and valley detections with an expected width of 25 seconds.

### Human samples

#### Children’s Hospital of Philadelphia

Subjects were recruited at the Children’s Hospital of Philadelphia (CHOP) from September 2015 to April 2018, and informed consent was acquired (IRB approval number 15-011817), as previously described (6). Groups included healthy children (HC), children with IBD (IBD), and children with IBD and concurrent CDI (IBD + CDI). Healthy children were age matched to those with IBD + CDI. Inclusion and exclusion criteria were described previously (6, 13). Untargeted metabolomics on the stool samples from these patients was performed as previously described (6). No images of human subjects are included in the figures, extended data, or supplementary materials.

#### University of Pittsburgh Medical Center

Adult patients with toxin-producing CDI were identified through the Enhanced Detection System for Healthcare-Associated Transmission (EDS-HAT) project (54). The Institutional Review Board of the University of Pittsburgh gave ethical approval for this work under Protocol STUDY21040126. Stool samples were stored at −80°C. Approximately 10 uL of each stool sample was thawed and streaked onto bile esculin azide (BEA) agar, and plates were incubated at 30°C for 48 hours. Individual colonies were isolated from plates that showed growth of presumed enterococci (i.e., white, gray, or black colonies that turned the agar underneath them black). Colonies were restreaked onto BEA agar before being stored at −80°C in tryptic soy broth with 16% glycerol.

### Whole genome sequencing and genomic analyses

#### Transposon mutant confirmation

Genomic DNA was extracted from the *E. faecalis* isolates using the Qiagen DNeasy Blood and Tissue kit. Sequencing library preparation and sequencing on the Illumina platform were performed by the CHOP Microbiome Center Sequencing Core. The whole-genome Average Nucleotide Identity was calculated, and the conserved regions between wild-type genome and cydA transposon genome were visualized using FastANI v1.34 (55). The genome alignment between wild-type and cydA transposon genomes was analyzed using mummer4 (56). The genomes were annotated through Bakta v1.8.2 (57) and SNPs between genomes were called using Snippy v4.6.0 (58). Mauve v2.4.0 was used to arrange the contigs of the genomes using complete *E. faecalis* OG1RF genome assembly (accession number: NC_017316) as the reference (59). The genome alignment was visualized using BLAST Ring Image Generator v0.95 (60). R package gggenes was used to better visualize the insertion of transposon in the genes (61).

#### Virulence factor analysis

Genome DNA was extracted from *E. faecalis* isolates using a Qiagen Dneasy Blood and Tissue Kit. Sequencing library preparation and sequencing on the Illumina platform were performed by SeqCenter, LLC, in Pittsburgh, PA. Genomes were *de novo* assembled using CLC Genomics Workbench v11.0.1. Fasta files of assembled genomes were analyzed using ResFinder and VirulenceFinder via the Center for Genomic Epidemiology (62–66).

### Isolate growth analysis

*E. faecalis* isolates were grown overnight in brain heart infusion (BHI) media and were then diluted 1:1,000 into fresh BHI or BHI supplemented with 10 μM hemin. Following overnight aerobic growth at 37°C with shaking at 170 rpm, the optical density at 600 nm (OD600) was measured for each culture. Three replicates of each isolate were tested, and OD600 values were background subtracted and averaged across replicates.

## Data Availability

WGS data for *E. faecalis* isolates have been deposited in NCBI under BioProject PRJNA996476 with accession numbers listed in Table S1. WGS data for *E. faecalis* transposon mutants have been deposited in NCBI under BioProject PRJNA1039837.

## SUPPLEMENTAL MATERIAL

Fig. S1 mbio.01656-23-s0001.tif Circle maps.

Fig. S2 mbio.01656-23-s0002.tif Ratio of *E. faecalis* OG1RF or *E. faecalis katA*::Tn CFU burdens to endogenous enterococcus CFU burdens.

Fig. S3 mbio.01656-23-s0003.tif Weight loss and clinical sickness scores of infected mice.

Fig. S4 mbio.01656-23-s0004.tif Relative abundance of heme in stools.

Fig. S5 mbio.01656-23-s0005.tif Core genome phylogeny of 25 *E. faecalis* isolates collected from CDI patient stools.

Legends mbio.01656-23-s0006.docx Supplemental figure legends.

Table S1 mbio.01656-23-s0007.docx Strains.

Table S2 mbio.01656-23-s0008.docx Liquid chromatography and mass spectrometry parameters used in the analysis of heme.

ASM does not own the copyrights to Supplemental Material that may be linked to, or accessed through, an article. The authors have granted ASM a non-exclusive, world-wide license to publish the Supplemental Material files. Please contact the corresponding author directly for reuse.
