# Supplementary figures and images for: Liberation of host heme by Clostridioides difficile-mediated damage enhances Enterococcus faecalis fitness during infection
Source: mBio. 2023 Dec 11;15(1):e01656-23. doi: 10.1128/mbio.01656-23 (PMC10790701; doi:10.1128/mbio.01656-23)

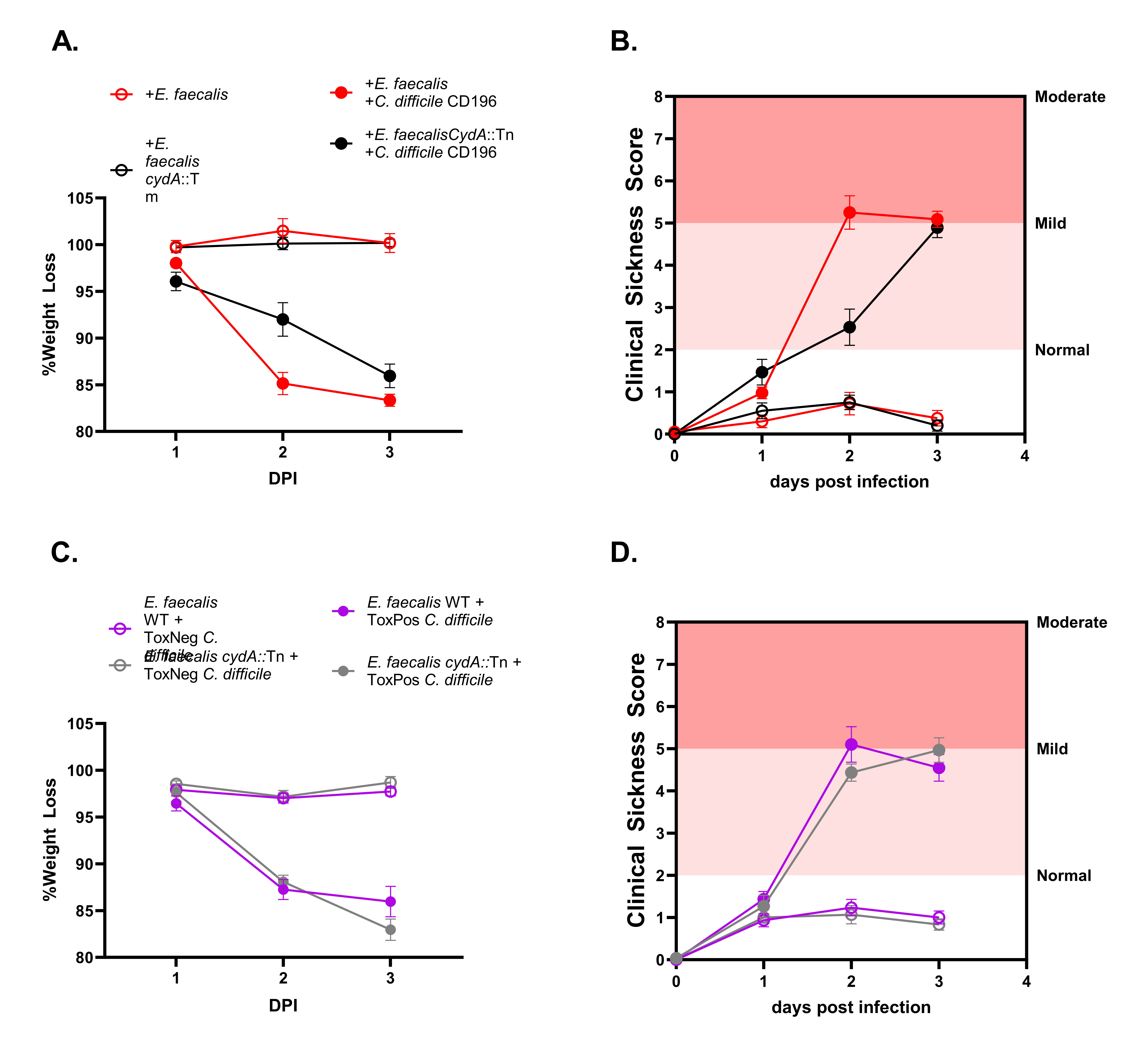

Supplement: Fig. S3 — Weight loss and clinical sickness scores of infected mice. [file mbio.01656-23-s0003.tif]

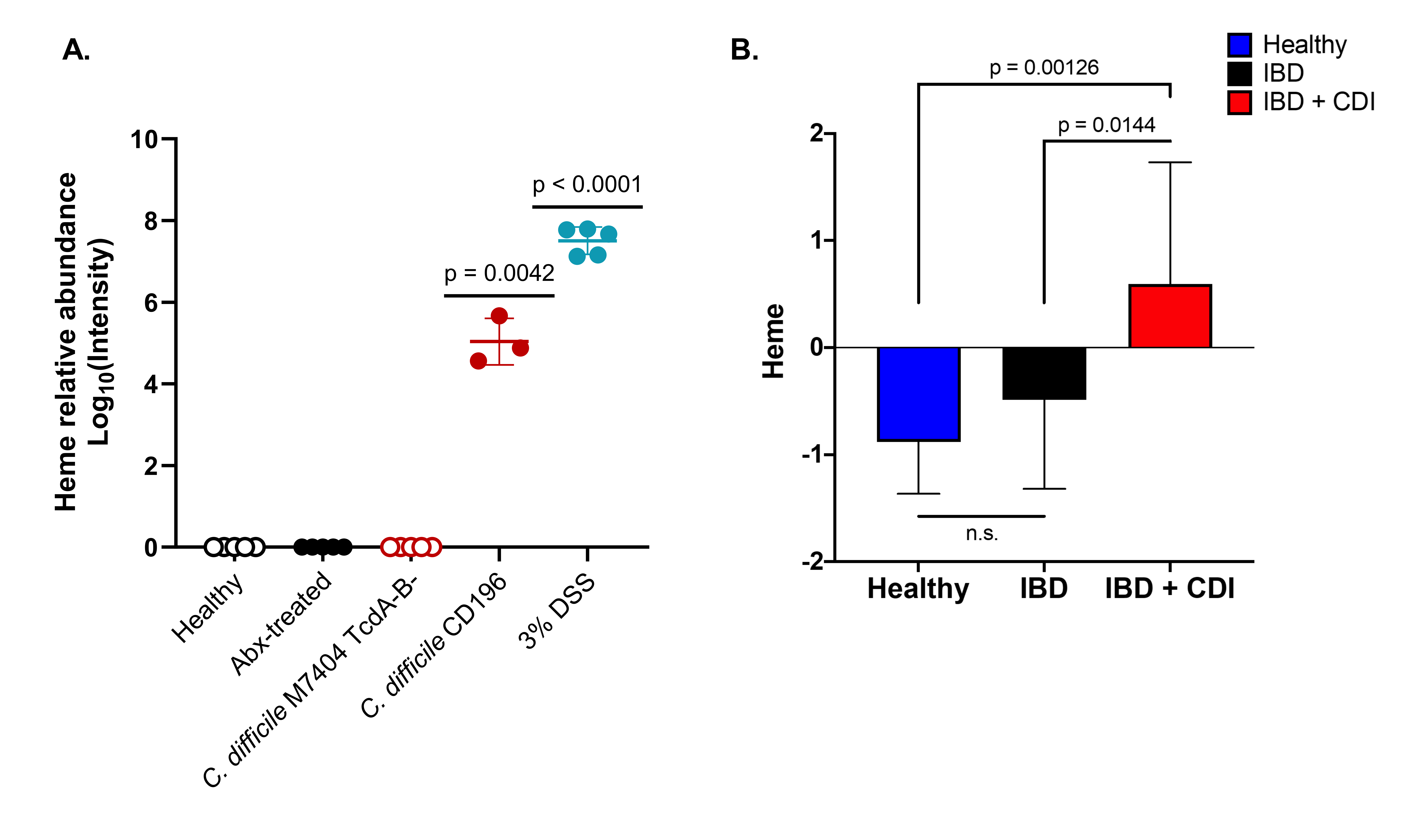

Supplement: Fig. S4 — Relative abundance of heme in stools. [file mbio.01656-23-s0004.tif]
