# Supplementary material for: Liberation of host heme by Clostridioides difficile-mediated damage enhances Enterococcus faecalis fitness during infection
Source: mBio. 2023 Dec 11;15(1):e01656-23. doi: 10.1128/mbio.01656-23 (PMC10790701; doi:10.1128/mbio.01656-23)
Supplement: Legends — Supplemental figure legends. [file mbio.01656-23-s0006.docx]

**SUPPLEMENTAL MATERIALS – FIGURE LEGENDS**

**Supplemental Figure 1**

**(A)** Circle map displaying an alignment of *E. faecalis* OG1RF and *E. faecalis* *cydA*::Tn genomes. Alignment displays 100% sequence identity across the entire genome except for the transposon inserted within the *cydA* gene. **(B)** Circle map displaying an alignment between *E. faecalis* OG1RF and *E. faecalis* *katA*::Tn genomes. Alignment displays 100% sequence identity across the entire genome except for the transposon inserted within the *katA* gene. **(C-D)** CFUs of the endogenous *Enterococcus* population in CDI mice **(C)** and non-CDI mice **(D)**. Data are shown with median; n=15 per group per day; *P*-values are from multiple Mann-Whitney test for significance with Bonferroni-Dunn correction for multiple comparisons. ns = not significant. LOD = limit of detection. DPI = days post-infection.

**Supplemental Figure 2**

Ratio of *E. faecalis* OG1RF or *E. faecalis katA*::Tn CFU burdens to endogenous enterococci CFU burdens in the stool of mice treated with cefoperazone and vancomycin and gavaged with each exogenous *E. faecalis* strain immediately prior to *C. difficile* infection, or without *C. difficile* infection. Data are shown with median; n=5 mice per group per day; significance was assessed with multiple Mann-Whitney test for significance with Bonferroni-Dunn correction for multiple comparisons. ns = not significant. LOD = limit of detection. Box plots show minimum, maximum, median, and interquartile range. DPI = days post-infection.

**Supplemental Figure 3**

**(A)** Weight loss of mice infected with *C. difficile* strain CD196. Data shown are mean ± SEM; n=10 +*E. faecalis* and +*E. faecalis cydA*::Tn, n=20 +*E. faecalis* +*C. difficile* CD196, n=15 +*E. faecalis cydA*::Tn +*C. difficile* CD196. **(B)** Clinical sickness score, a composite score of weight loss, stool consistency, and behavior, from mice infected with *C. difficile* strain CD196. Data shown are mean ± SEM; n=10 +*E. faecalis* and +*E. faecalis cydA*::Tn, n=20 +*E. faecalis* +*C. difficile* CD196, n=15 +*E. faecalis cydA*::Tn +*C. difficile* CD196. **(C)** Weight loss of mice infected with *C. difficile* strain M7404. Data shown are mean ± SEM; n=15 for all groups. **(D)** Clinical sickness score from mice infected with *C. difficile* strain M7404. Data shown are mean ± SEM; n=15 for all groups. Significance measured with Welch’s *t* test, * = *P* < 0.0001

**Supplemental Figure 4**

**(A)** Relative abundance of heme in the stool of mice quantified by UHPLC-HRMS at day 3 of CDI or day 6 of DSS treatment (mean± s.d., n=5/group, n=3 for CD196, significance measured by one sample *t* test against a hypothetical value of 0) **(B)** Relative abundance of heme in the stool of pediatric patients with IBD and *C. difficile* infection (IBD + CDI) (mean ± s.d., n= 15) versus patients with just IBD (mean ± s.d., n= 27) or healthy controls (mean ± s.d., n= 36, one-sides Welch ANOVA).

**Supplemental Figure 5**

Core genome phylogeny of 25 *E. faecalis* isolates collected from CDI patient stools. Isolate name, multi-locus sequence type (ST) and presence of relevant genetic features (filled boxes) are shown to the right of the phylogeny. SLV = single locus variant. Purple = *cydABDC* operon; Green = acquired antibiotic resistance genes identified by ResFinder; Yellow = plasmid *rep* genes identified by PlasmidFinder; Blue = *E. faecalis* virulence factors identified by VirulenceFinder.
