# Supplementary material for: Liberation of host heme by Clostridioides difficile-mediated damage enhances Enterococcus faecalis fitness during infection
Source: mBio. 2023 Dec 11;15(1):e01656-23. doi: 10.1128/mbio.01656-23 (PMC10790701; doi:10.1128/mbio.01656-23)
Supplement: Table S1 — Strains. [file mbio.01656-23-s0007.docx]

**SUPPLEMENTARY TABLE 1 – Strains used in this study**

| Bacterial Strain | Genotype | Source | BioSample |
| --- | --- | --- | --- |
| *Clostridioides difficile* CD196 | Ribotype 027 | ^67^ |  |
| *Clostridioides difficile* M7404 | Ribotype 027 | ^68^ |  |
| *Clostridioides difficile* M7404 TcdA^-^ TcdB^-^ | Toxin knockout | ^68^ |  |
| *Enterococcus faecalis* OG1RF |  | ^69^ | [SAMN38228696](https://www.ncbi.nlm.nih.gov/biosample/38228696) |
| *Enterococcus faecalis* OG1RF *cydA::*Cm | Transposon mutant in *cydA* | ^69^ | [SAMN38228697](https://www.ncbi.nlm.nih.gov/biosample/38228697) |
| *Enterococcus faecalis* OG1RF *katA*::Cm | Transposon mutant in *katA* | ^69^ | [SAMN38228698](https://www.ncbi.nlm.nih.gov/biosample/38228698) |
| *Enterococcus faecalis* DVT1895 |  | Clinical Isolate (University of Pittsburgh) | [SAMN36574728](https://www.ncbi.nlm.nih.gov/biosample/36574728) |
| *Enterococcus faecalis* DVT1896 |  | Clinical Isolate (University of Pittsburgh) | [SAMN36574729](https://www.ncbi.nlm.nih.gov/biosample/36574729) |
| *Enterococcus faecalis* DVT1897 |  | Clinical Isolate (University of Pittsburgh) | [SAMN36574730](https://www.ncbi.nlm.nih.gov/biosample/36574730) |
| *Enterococcus faecalis* DVT1898 |  | Clinical Isolate (University of Pittsburgh) | [SAMN36574731](https://www.ncbi.nlm.nih.gov/biosample/36574731) |
| *Enterococcus faecalis* DVT1899 |  | Clinical Isolate (University of Pittsburgh) | [SAMN36574732](https://www.ncbi.nlm.nih.gov/biosample/36574732) |
| *Enterococcus faecalis* DVT1900 |  | Clinical Isolate (University of Pittsburgh) | [SAMN36574733](https://www.ncbi.nlm.nih.gov/biosample/36574733) |
| *Enterococcus faecalis* DVT1901 |  | Clinical Isolate (University of Pittsburgh) | [SAMN36574734](https://www.ncbi.nlm.nih.gov/biosample/36574734) |
| *Enterococcus faecalis* DVT1902 |  | Clinical Isolate (University of Pittsburgh) | [SAMN36574735](https://www.ncbi.nlm.nih.gov/biosample/36574735) |
| *Enterococcus faecalis* DVT1903 |  | Clinical Isolate (University of Pittsburgh) | [SAMN36574736](https://www.ncbi.nlm.nih.gov/biosample/36574736) |
| *Enterococcus faecalis* DVT1904 |  | Clinical Isolate (University of Pittsburgh) | [SAMN36574737](https://www.ncbi.nlm.nih.gov/biosample/36574737) |
| *Enterococcus faecalis* DVT1905 |  | Clinical Isolate (University of Pittsburgh) | [SAMN36574738](https://www.ncbi.nlm.nih.gov/biosample/36574738) |
| *Enterococcus faecalis* DVT1906 |  | Clinical Isolate (University of Pittsburgh) | [SAMN36574739](https://www.ncbi.nlm.nih.gov/biosample/36574739) |
| *Enterococcus faecalis* DVT1907 |  | Clinical Isolate (University of Pittsburgh) | [SAMN36574740](https://www.ncbi.nlm.nih.gov/biosample/36574740) |
| *Enterococcus faecalis* DVT1908 |  | Clinical Isolate (University of Pittsburgh) | [SAMN36574741](https://www.ncbi.nlm.nih.gov/biosample/36574741) |
| *Enterococcus faecalis* DVT1909 |  | Clinical Isolate (University of Pittsburgh) | [SAMN36574742](https://www.ncbi.nlm.nih.gov/biosample/36574742) |
| *Enterococcus faecalis* DVT1910 |  | Clinical Isolate (University of Pittsburgh) | [SAMN36574743](https://www.ncbi.nlm.nih.gov/biosample/36574743) |
| *Enterococcus faecalis* DVT1911 |  | Clinical Isolate (University of Pittsburgh) | [SAMN36574744](https://www.ncbi.nlm.nih.gov/biosample/36574744) |
| *Enterococcus faecalis* DVT1912 |  | Clinical Isolate (University of Pittsburgh) | [SAMN36574745](https://www.ncbi.nlm.nih.gov/biosample/36574745) |
| *Enterococcus faecalis* DVT1913 |  | Clinical Isolate (University of Pittsburgh) | [SAMN36574746](https://www.ncbi.nlm.nih.gov/biosample/36574746) |
| *Enterococcus faecalis* DVT1914 |  | Clinical Isolate (University of Pittsburgh) | [SAMN36574747](https://www.ncbi.nlm.nih.gov/biosample/36574747) |
| *Enterococcus faecalis* DVT1915 |  | Clinical Isolate (University of Pittsburgh) | [SAMN36574748](https://www.ncbi.nlm.nih.gov/biosample/36574748) |
| *Enterococcus faecalis* DVT1916 |  | Clinical Isolate (University of Pittsburgh) | [SAMN36574749](https://www.ncbi.nlm.nih.gov/biosample/36574749) |
| *Enterococcus faecalis* DVT1919 |  | Clinical Isolate (University of Pittsburgh) | [SAMN36574750](https://www.ncbi.nlm.nih.gov/biosample/36574750) |
| *Enterococcus faecalis* DVT1920 |  | Clinical Isolate (University of Pittsburgh) | [SAMN36574751](https://www.ncbi.nlm.nih.gov/biosample/36574751) |
| *Enterococcus faecalis* DVT1930 |  | Clinical Isolate (University of Pittsburgh) | [SAMN36574752](https://www.ncbi.nlm.nih.gov/biosample/36574752) |

**REFERENCES**

67. Stabler RA, He M, Dawson L, Martin M, Valiente E, Corton C, Lawley TD, Sebaihia M, Quail MA, Rose G, Gerding DN, Gibert M, Popoff MR, Parkhill J, Dougan G, Wren BW. 2009. Comparative genome and phenotypic analysis of Clostridium difficile 027 strains provides insight into the evolution of a hypervirulent bacterium. Genome Biol 10:R102. <https://doi.org/10.1186/gb-2009-10-9-r102>.

68. Carter GP, Chakravorty A, Pham Nguyen TA, Mileto S, Schreiber F, Li L, Howarth P, Clare S, Cunningham B, Sambol SP, Cheknis A, Figueroa I, Johnson S, Gerding D, Rood JI, Dougan G, Lawley TD, Lyras D. 2015. Defining the roles of TcdA and TcdB in localized gastrointestinal disease, systemic organ damage, and the host response during Clostridium difficile infections. mBio 6:e00551. <https://doi.org/10.1128/mBio.00551-15>.

69. Dale JL, Beckman KB, Willett JLE, Nilson JL, Palani NP, Baller JA, Hauge A, Gohl DM, Erickson R, Manias DA, Sadowsky MJ, Dunny GM. 2018. Comprehensive functional analysis of the Enterococcus faecalis core genome using an ordered, sequence-defined collection of insertional mutations in strain OG1RF. mSystems 3:e00062-18. <https://doi.org/10.1128/mSystems.00062-18>.
