# Supplementary material for: Liberation of host heme by Clostridioides difficile-mediated damage enhances Enterococcus faecalis fitness during infection
Source: mBio. 2023 Dec 11;15(1):e01656-23. doi: 10.1128/mbio.01656-23 (PMC10790701; doi:10.1128/mbio.01656-23)
Supplement: Table S2 — Liquid chromatography and mass spectrometry parameters used in the analysis of heme. [file mbio.01656-23-s0008.docx]

**SUPPLEMENTARY TABLE 2 – Liquid chromatography and mass spectrometry parameters used in the analysis of heme**

| HPLC conditions | | HESI-MS conditions | |
| --- | --- | --- | --- |
| Column dimensions | 100 x 2.1 mm | Ionization mode | Positive ESI |
| Stationary phase | C18-PFP | Capillary voltage | 3.5 kV |
| Particle size | 2.0 µm | Capillary temperature | 325 °C |
| Column temperature | 50 °C | Aux gas temperature | 375 °C |
| Mobile Phase A | Water + 0.1% FA | Aux gas flow rate | 10 |
| Mobile Phase B | Methanol | Sheath gas flow rate | 55 |
| Flow rate | 0.350 mL/min | Sweep gas flow rate | 1 |
| Injection volume | 4 µL | S-lens RF level | 100 |
| Autosampler temperature | 5 °C | Resolution | 17,500 |
|  |  | Scan range | 70.0 to 1,000 *m/z* |
|  |  | AGC target | 3e6 |
|  |  | Max inject time | 100 ms |
| Gradient |  |  |  |
| Time | Flow [mL/min] | %A | %B |
| 0 | 0.35 | 100 | 0 |
| 3 | 0.35 | 100 | 0 |
| 8 | 0.35 | 0 | 100 |
| 10.5 | 0.35 | 0 | 100 |
| 10.7 | 0.35 | 100 | 0 |
| 11 | 0.6 | 100 | 0 |
| 13 | 0.6 | 100 | 0 |
| 13.5 | 0.35 | 100 | 0 |
